# Encephalitis Unraveled: The Unlikely Encounter of Sickle Cell Disease and Cerebral Malaria in a Teenager

**DOI:** 10.3390/diagnostics15121470

**Published:** 2025-06-10

**Authors:** Christer Ruff, Leonie Zerweck, Andrea Bevot, Jonathan Remppis, Benjamin Bender, Ulrike Ernemann, Georg Gohla

**Affiliations:** 1 Department of Diagnostic and Interventional Neuroradiology, Eberhard Karls-University Tübingen, Hoppe-Seyler-Str. 3, 72076 Tübingen, Germanygeorg.gohla@med.uni-tuebingen.de (G.G.); 2 Department of Pediatric Neurology, University Hospital Tübingen, Hoppe-Seyler-Str. 3, 72076 Tübingen, Germany; 3 German Center for Infection Research (DZIF), Partner Site Tübingen, 72076 Tübingen, Germany

**Keywords:** central nervous system (CNS), cerebral malaria, hemoglobin (HB), magnetic resonance imaging (MRI), sickle cell disease (SCD)

## Abstract

Sickle-cell disease (SCD) is a group of inherited blood disorders in which a mutation in the β-globin (HBB) gene causes red blood cells to produce abnormal hemoglobin, known as Hb S. SCD is characterized by an autosomal-recessive pattern of inheritance, implying that for a child to manifest the condition, they must inherit an Hb S allele from both parents (HbSS) or one Hb S allele and another β-globin variant, such as Hb C or β-thalassemia (HbSC, HbS/β-thal). It has been observed that (heterozygote) carriers of one copy of the sickle-cell trait (HbAS) are typically healthy and can even gain partial protection from severe malaria. The term “severe and complicated malaria” is delineated based on specific clinical and laboratory characteristics in the presence of *Plasmodium falciparum* parasitemia. The prevalent forms of severe malaria among African children include cerebral malaria, respiratory distress, and severe malaria anemia. Cerebral malaria is a rare complication of malaria infection and is associated with a high mortality rate.

**Figure 1:**
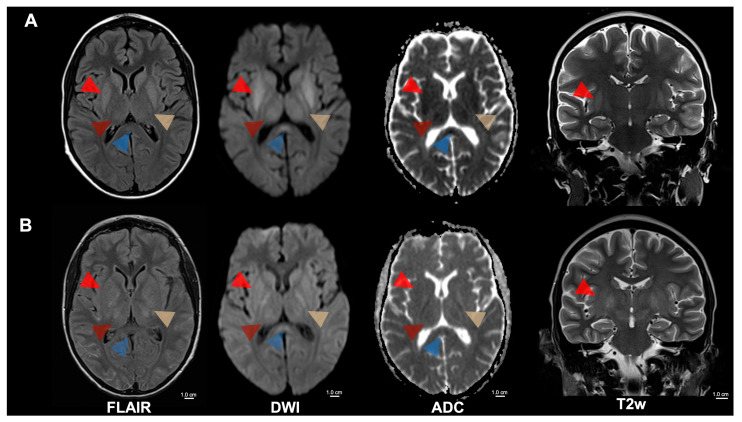
MRI of cerebral malaria of a 15-year-old patient with sickle cell disease, experiencing recurrent hemolytic crises, presented in a stuporous state necessitating intensive care. Focal and generalized seizures, along with left-sided motor issues, prompted an investigation revealing *Plasmodium falciparum* malaria. (**A**) Initial MRI showed symmetrical T2 signal increase and diffusion restriction of the internal capsule (yellow arrowhead), the basal ganglia (bright red arrowhead), the thalamus (dark red arrowhead), the mesencephalon, the subthalamic nuclei, and the splenium corporis callosi (blue arrowhead). Furthermore, a discrete diffuse T2 signal increase was noticed in the cerebellum and cortical areas of the central region. (**B**) A follow-up MRI after 5 days revealed decreased diffusion restriction in the areas mentioned above but an increasingly swollen aspect. Severe and complicated malaria is defined based on specific clinical and laboratory features in the presence of *Plasmodium falciparum* parasitemia. The most typical forms of severe malaria are cerebral malaria, respiratory distress (caused by deep or acidotic breathing), and severe malaria anemia [1,2]. Cerebral malaria carries significant mortality and morbidity. Its pathophysiology involves parasitized red blood cells lodging within cerebral capillaries, a surge of cytokine release, buildup of immune cells and platelets, and the shedding of microparticles. Together, these events compromise the blood–brain barrier and produce consequential brain injury. The severity of this phenomenon is reflected in neurological findings, ranging from simple delirium to profound coma. Sickle-cell disease (SCD) comprises inherited hemoglobinopathies caused by a mutation in the β-globin (HBB) gene that yields the abnormal hemoglobin variant Hb S. Individuals who carry a single copy of this mutation (HbAS heterozygotes) are usually healthy and enjoy partial protection against severe malaria, whereas homozygote people with SCD face an elevated risk of developing severe malarial illness [3]. Potchen et al. conducted a study of acute MRI scans from 152 children with proven cerebral malaria and found that abnormalities were most prevalent in those who also showed malarial retinopathy [4,5]. The typical findings included pronounced cerebral swelling, elevated T2 signal, diffusion-weighted imaging changes in cortical regions, deep gray nuclei, and white-matter tracts. The basal ganglia were most frequently affected, exhibiting T2-hyperintensity that ranged from mild to marked, often accompanied by swelling and mass effect. Nickerson et al. described MRI appearances with widespread petechial hemorrhages at the gray-white junction, within the corpus callosum, and along the internal capsules on susceptibility-weighted imaging—changes that correspond to histological ring microhemorrhages [6]. Furthermore, edema and multifocal T2-hyperintensities in the corpus callosum and posterior limbs of the internal capsules were also observed, suggesting ischemia resulting from microvascular blockage by clumped *Plasmodium falciparum*-infected erythrocytes. The degree of such sequestration has been quantitatively linked to premortem coma in cerebral malaria patients [7]. FLAIR = Fluid-attenuated inversion recovery; DWI = Diffusion-weighted magnetic resonance imaging; ADC = Apparent diffusion coefficient; T2w = T2-weighted; cm = centimeter.

**Figure 2:**
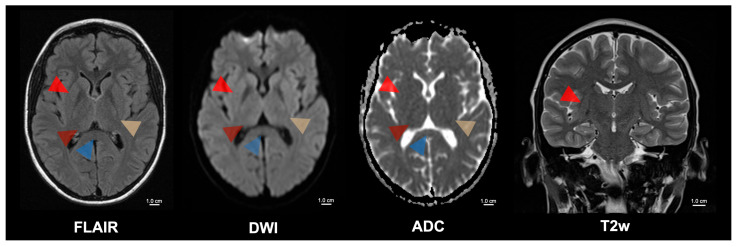
Five-month follow-up of the patient with cerebral malaria. Cerebral malaria is associated with a high mortality rate. However, those patients who survive often have a complete recovery with minimal or no long-term sequelae. Despite the formerly extensive MRI changes, the patient had a complete motor and cognitive recovery after treatment with artesunate and artemether/lumefantrine. The follow-up MRI of the presented patient showed a complete regression of the previously observed pathological signal changes in the brain parenchyma. These signal changes were formerly present in the internal capsule (yellow arrowhead), the basal ganglia (bright red arrowhead), the thalamus (dark red arrowhead), the mesencephalon, the subthalamic nuclei, and the splenium corporis callosi (blue arrowhead). Supplementary Table S1 contains an overview of the patient’s progress over time, including the date of infection, the start of treatment, the antimalarial drugs used (artesunate and artemether/lumefantrine), the dosages and the date of cure. Rasalkar et al. detailed four cases in which MRI consistently revealed bilateral thalamic infarctions, with or without hemorrhage; one patient additionally had acute hemorrhagic infarctions in the brainstem and cerebellum and subsequently died, whereas the other three survived with antimalarial therapy, and follow-up scans showed resolution of the thalamic lesions [8]. In patients returning from malaria-endemic regions, any abrupt neurological deterioration must prompt an immediate malaria diagnosis, as malaria treatment has been shown to reverse changes in motor and cognitive function, and reverse striking MRI abnormalities, thereby allowing full neurological recovery in some cases. FLAIR = Fluid-attenuated inversion recovery; DWI = Diffusion-weighted magnetic resonance imaging; ADC = Apparent diffusion coefficient; T2w = T2-weighted; cm = centimeter.

## Data Availability

All relevant data are contained within the article/Supplementary Material.

## Supplementary Materials

The following supporting information can be downloaded at https://www.mdpi.com/article/10.3390/diagnostics15121470/s1, Table S1: Patient’s timeline including the date of infection, treatment initiation, antimalarial drugs used, dosages, and the date of cure.

## Abbreviations

The following abbreviations are used in this manuscript:

ADC	Apparent diffusion coefficient
DWI	Diffusion-weighted magnetic resonance imaging
FLAIR	Fluid-attenuated inversion recovery
HB	Hemoglobin
MRI	Magnetic resonance imaging
SCD	Sickle cell disease
