# Supplementary material for: Encephalitis Unraveled: The Unlikely Encounter of Sickle Cell Disease and Cerebral Malaria in a Teenager
Source: Diagnostics (Basel). 2025 Jun 10;15(12):1470. doi: 10.3390/diagnostics15121470 (PMC12192274; doi:10.3390/diagnostics15121470)
Supplement: Supplementary file 1 [file diagnostics-15-01470-s001.zip › diagnostics-3630096-supplementary.pdf]

Supplementary Table S1. Patient's timeline including the date of infection, treatment initiation, antimalarial drugs used, dosages, and the date of cure.

Date / Period	Event / Intervention	Brief Comment / Outcome
Age of 5	Migration from Nigeria to Germany.	
Age 5 – 15 years	Hydroxyurea and voxelotor treatment. Multiple sickle cell crises and RBC transfusions.	Long-term disease-modifying treatment for sickle-cell disease.
12th November 2023	Return from West Africa.	History of 3-month stay in Benin & Lagos. No malaria chemoprophylaxis.
16th – 19th November 2023	Hemolytic crisis without fever.	Minimal hemoglobin 5.4 g/dl. RBC transfusion. Clinical improvement and hospital discharge after 3 days.
1st – 7th December 2023	Hemolytic crisis and fever. Presumed autoimmune hemolysis.	Minimal hemoglobin 3.9 g/dl. RBC transfusion. Detection of RBC allo-auto-antibodies. Initiation of Methylprednisolone and Azathioprine treatment. Broad-spectrum antibiotic treatment. Clinical improvement and hospital discharge after 6 days.
11th – 15th December 2023	Hemolytic crisis and fever.	Multiple RBC transfusions. Rituximab treatment. Broad-spectrum antibiotic treatment. Hospital admission.
15th – 19th December 2023	Progressive neurological deterioration.	cMRI and EEG abnormalities. Lumbar puncture without abnormal findings. Intravenous immunoglobulins. Eculizumab treatment. Escalation of antibiotic treatment. Admission to intermediate care ward.
20th December 2023	Recurrent seizures, Coma. Additional pulmonary decline.	Initiation of anticonvulsive treatment. Admission to intensive care unit. Intubation and mechanical ventilation.
21st December 2023	Microscopic detection of *Plasmodium falciparum*. Immediate treatment with artesunate i.v..	Pre-treatment parasitemia 7.923/µl. Artesunate 84 mg (2.4 mg/kg) administered by i.v. injection at 0, 12, and 24 hours.
23rd December 2023	Quick clinical improvement and reduction of parasitemia. Switch to artemether/lumefantrine p.o..	Artemether/lumefantrine 80mg/480mg administered 2 times daily over 3 days. Extubation.
24th – 27th December 2023	Residual left-sided motor deficit (leg-predominant), gradually improving.	Trend towards recovery. Transfer to regular ward.
27th December 2023 – 5th January 2024	Stable parasite clearance. Complete motor & cognitive recovery. No further seizures.	Discharge from the hospital on 5th January 2024.

RBC = red blood cells; cMRI = cerebral magnetic resonance imaging; EEG, electroencephalography; i.v. = intravenous; p.o. = per os
